# Another Iconoclast

**Published:** 1892-11

**Authors:** 


### ANOTHER ICONOCLAST.

Some time ago, Prof. H. C. Wood pointed out that it is all a mistake about chlorate of potassa oxidizing the blood;—thus destroying one of the favorite delusions of the profession,—a theory or belief upon which was founded a very popular treatment for many diseases. The JOURNAL commented on it at the time. Now comes Prof. Hare, who knocks the props from under several other popular *beliefs*, and leaves us nothing to stand on in several emergencies. For instance, he says (Address in Medicine, *Lancet-Clinic*, Nov. 5), that in respiratory failure during anæsthesia, it is all wrong to use the Farradic current, with one pole on the neck, over the phrenic nerve, the other over the diaphragm. * * * "What we desire nnder such circumstances is a slow contraction and relaxation of the diaphragm, such as we see in health, and the nearest approach to this is, theoretically, to be obtained by the current which is slowly interrupted. Practically, however, we find that both of these currents are worthless, and worse than worthless,—are dangerous." Dr. Hare also says that the practice of inverting, partly or completely, the patient in respiratory or circulating failure during anæsthesia is wrong, "It goes without saying," says the author, that this is only justifiable, when heart failure is shown by marked facial pallor. If the respiration is at fault we should carefully avoid any inversion, because the presence of still more venous blood about the already exhausted respiratory centre can not aid it, but only injure it." It is also all wrong, according to Dr. Hare, to give a hypodermic of morphia, as is generally done, in hemorrhage of the lungs, "to allay nervousness of the patient." The Doctor gives his reasons for all these objections, and they appear to be logical. In this latter breaking of a favorite idol, he says: "While it (the morphia) may allay nervousness, it will likewise cause an increased flow of blood and increase the loss of this fluid."

Thus, as medicine progresses, we have to unlearn many things. Even opium, the almost universal panacea, and the stand-by for years, is falling from grace, with the advent of the newer pathology. Dr. Hare says, "As an interesting point in the evolution of medicine, we find the field of usefulness of opium constantly growing smaller, and in no direction has it become more circumscribed than in the treatment of diarrhœal disturbances. * * Opium may be given for pain, but not for diarrhœa."

And thus it goes. What, with the assurance that William Tell was a myth; that prohibition does not prohibit; that alcohol, the universal and indispensible *stimulant*, does not stimulate; that chlorate potassa does not oxidize the blood, etc., etc., what is one to believe?

